# Non-syndromic occurrence of true generalized microdontia with mandibular mesiodens - a rare case

**DOI:** 10.1186/1746-160X-7-19

**Published:** 2011-10-28

**Authors:** Seema D Bargale, Shital DP Kiran

**Affiliations:** 1 Department of Pedodontics and Preventive Dentistry, AECS Maruthi Dental College and Research Center, India

**Keywords:** Generalised microdontia, Hyperdontia, Permanent dentition, Mandibular supernumerary tooth

## Abstract

Abnormalities in size of teeth and number of teeth are occasionally recorded in clinical cases. True generalized microdontia is rare case in which all the teeth are smaller than normal. Mesiodens is commonly located in maxilary central incisor region and uncommon in the mandible. In the present case a 12 year-old boy was healthy; normal in appearance and the medical history was noncontributory. The patient was examined and found to have permanent teeth that were smaller than those of the average adult teeth. The true generalized microdontia was accompanied by mandibular mesiodens. This is a unique case report of non-syndromic association of mandibular hyperdontia with true generalized microdontia.

## Introduction

Microdontia is a rare phenomenon. The term microdontia (microdentism, microdontism) is defined as the condition of having abnormally small teeth [1]. According to Boyle, "in general microdontia, the teeth are small, the crowns short, and normal contact areas between the teeth are frequently missing" [2] Shafer, Hine, and Levy [3] divided microdontia into three types: (1) Microdontia involving only a single tooth; (2) relative generalized microdontia due to relatively small teeth in large jaws and (3) true generalized microdontia, in which all the teeth are smaller than normal. According to these authors, aside from its occurrence in some cases of pituitary dwarfism, true generalized microdontia is exceedingly rare. Microdontia of a single tooth can be further classified into (1) microdontia of the whole tooth, (2) microdontia of the crown of the tooth, and (3) microdontia of the root alone [4].

Involvement of the entire dentition is rare and been reported in radiation or chemotherapeutic treatment during the developmental stage of the teeth [5], pituitary dwarfism [3] and Fanconi's anemia [6]. The syndromes associated with microdontia are Gorlin-Chaudhry-Moss syndrome, Williams's syndrome, Chromosome d/u, 45X [Ullrich-Turner syndrome], Chromosome 13[trisomy 13], Rothmund-Thomson syndrome, Hallermann-Streiff, Orofaciodigital syndrome (type 3), Oculo-mandibulo-facial syndrome, Tricho-Rhino-Phalangeal, type1 Branchio-oculo-facial syndrome.

Supernumerary teeth are defined as any supplementary tooth or tooth substance in addition to usual configuration of twenty deciduous and thirty two permanent teeth [7]. Classification of supernumerary teeth may be based on position or morphology. Positional variations include anterior mesiodens, para-premolars, para-molars and disto-molars. Variations in morphology consist of supplemental and rudimentary types [8].

Supernumerary teeth are common in the maxillary anterior region although supernumerary teeth have been reported in the incisor region of the mandible are very rare. Although supernumerary teeth have been reported in the incisor region of the mandible, they are very rare [9-14].

Conditions, in which supernumery teeth found, are cleidocranial dysplesia, cleft lip and cleft palate [15]. Syndromes associated with supernumery teeth are Familial adenomatous polyposis [Gardner's], Apert, Klippel-Trenaunay-Weber, Craniometaphyseal dysplasia, Trisomy 21[Down's], Nance-Horan, Orofaciodigital syndrome (type 3), Sturge-weber and Tricho-Rhino-Phalangeal, type1.

In the case described here is a bizarre generalized microdontia involving the entire dentition along with mandibular mesiodens without any other apparent systemic conditions.

## Case Report

The patient was a 12 year old boy, only child of consanguineous parents, reported to the department of pedodontics and preventive dentistry with the complaint of small teeth. Parents noted small teeth ever since the eruption of permanent teeth. No abnormalities were reported, however, in their extended family.

### Physical examination

Physical growth was within normal limits. The patient was of normal in stature, appearance, height, and weight for his age. Upon examination of the limbs, hands, skin, hair, nails and eyes were all appeared normal. No abnormality was noted in neck, back, muscles, cranium and joints as well. Intellectual and scholastic performance was also normal. His medical history was unremarkable; no other abnormalities were noted in the history apart from the difficult delivery. The child was examined and found to be free of any gross abnormalities.

His blood profile was normal. Serum calcium, phosphorous and alkaline phosphatase levels were also normal. Endocrinological investigation was carried out to rule in or out the possibility of hormonal disorder, and the results were within normal limits.

### Intraoral examination

**Figure 1:**
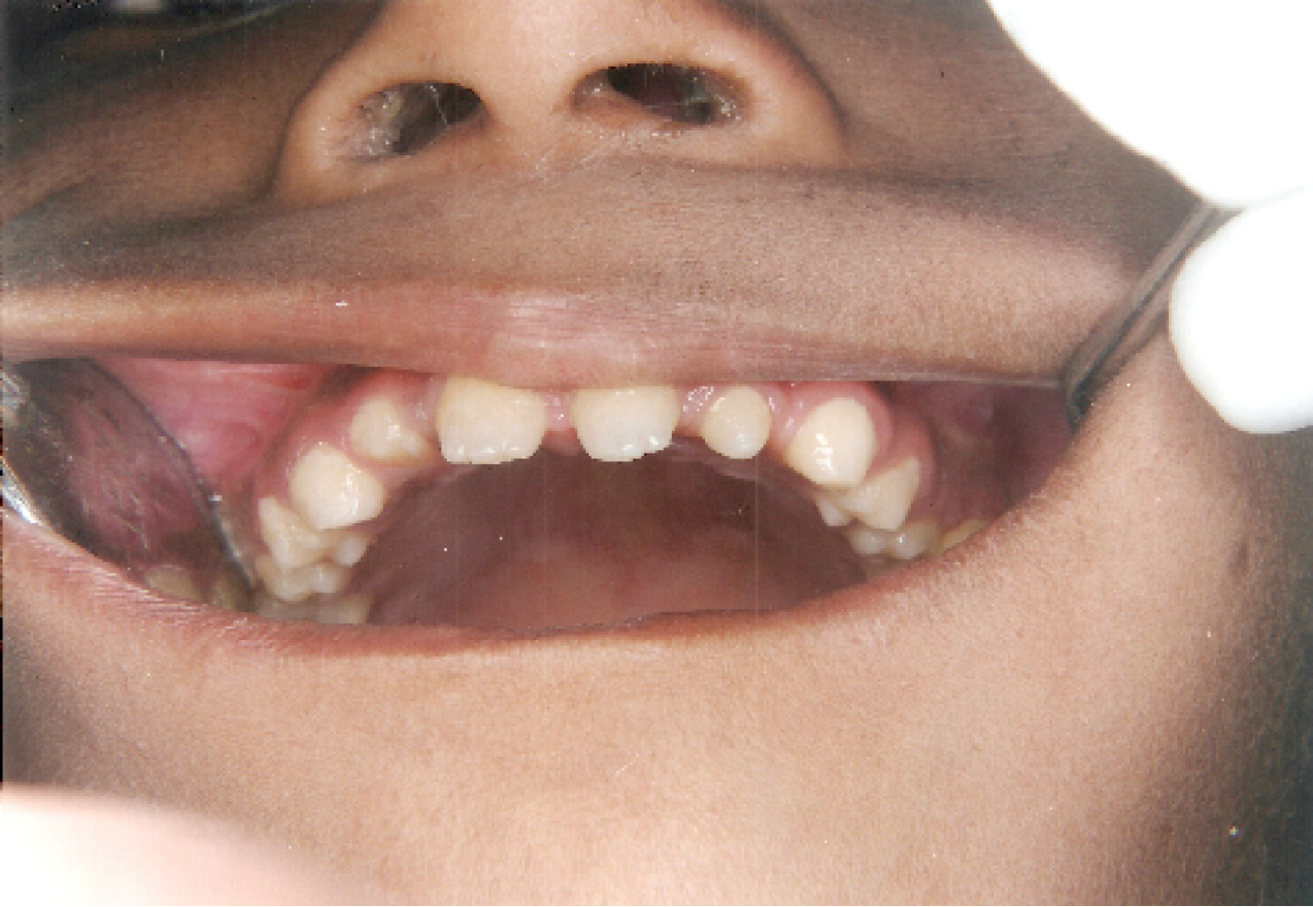
**Intra oral view of the upper arch**.

**Figure 2:**
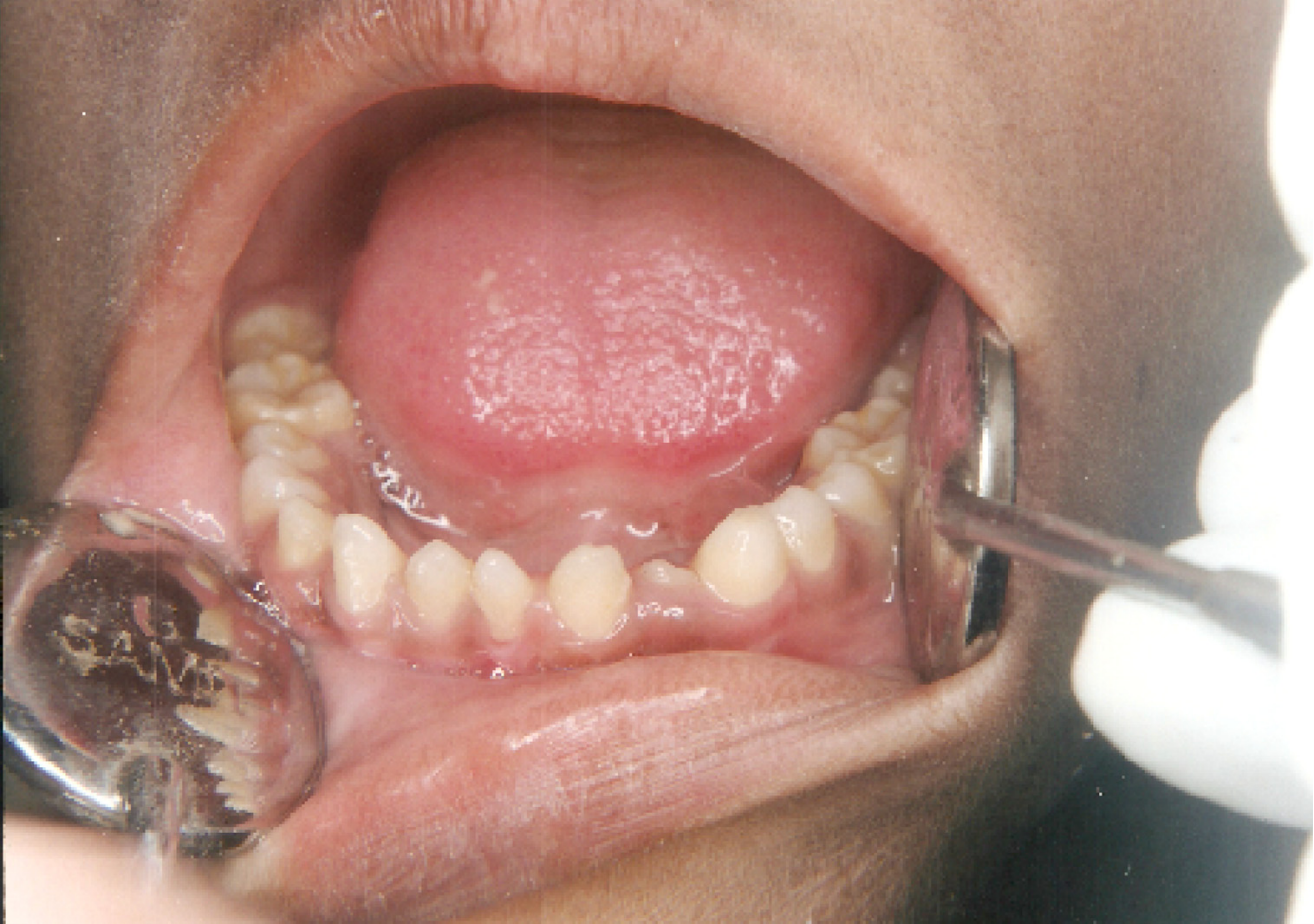
**Intra oral view of the lower arch**.

**Figure 3:**
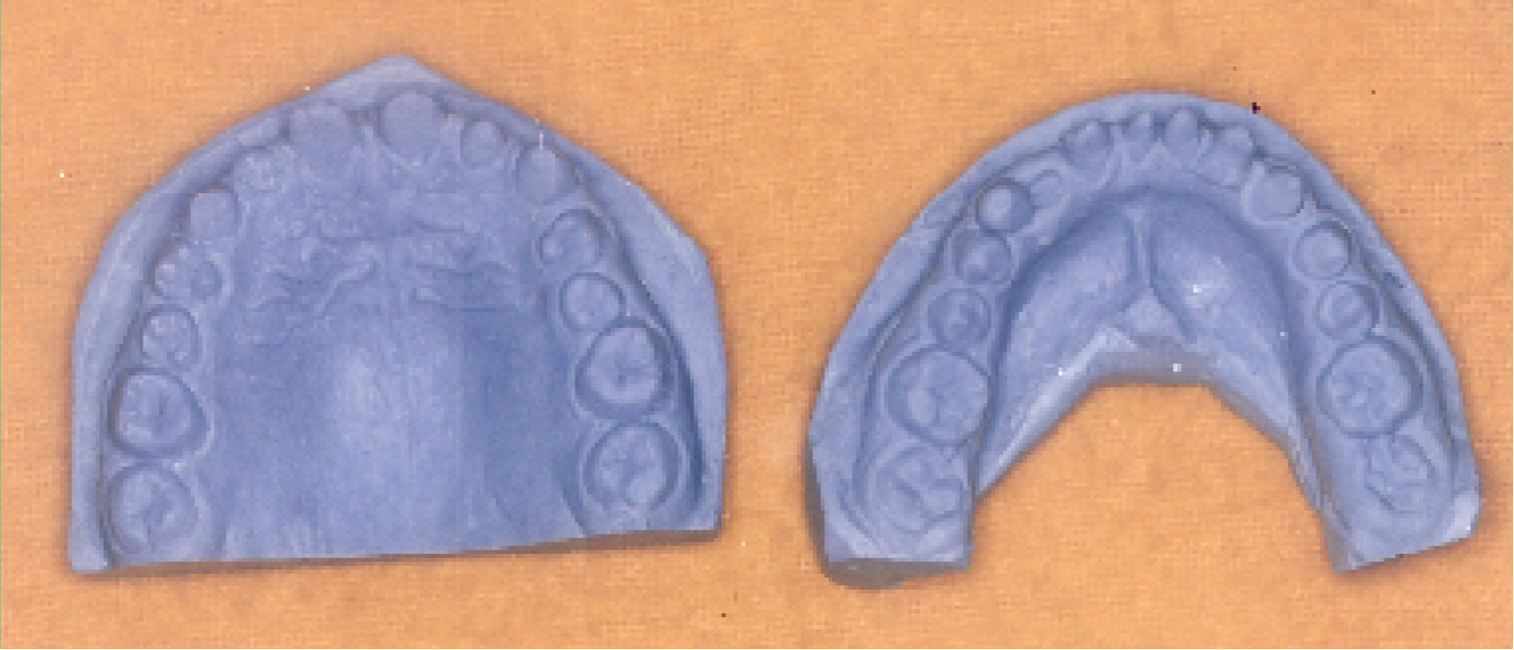
**Diagnostic casts showing the morphology of the teeth**.

The intraoral soft tissues were healthy, but the teeth were abnormal in size and shape (Figure 1 and 2). Diagnostic casts were obtained to aid in diagnosis (Figure 3). Patient was in permanent dentition, teeth present were small in size. The patient had normal occlusion with excessive spacing between the teeth. Fully erupted mandibular mesiodens was present between the central incisors.

The anterior teeth lacked normal size in all dimensions. Most of the anterior teeth were "peg-shaped" without the typical variation in mesiodistal and labiolingual dimensions. Almost all the maxillary anterior teeth did not have lingual pits whereas mandibular central and lateral incisors had prominent pits on the lingual surfaces. The posterior teeth were also small and exhibited a short occlusogingival dimension. Overall, the dentition was smaller than that of the average adult (Table 1 and 2). Orthopantomogram or the Intra oral periapical radigraph could not be taken because the patient was not able to afford.

**Table 1:** Comparison of buccolingual/labiolingual and mesiodistal crown dimensions with an anatomic average* of the right side maxillary and mandibular teeth

Right side	Central incisor	Lateral incisor	Canine	First premolar	Second premolar	First molar	Second molar	Total
Maxillary	MD LL	MD LL	MD LL	MD BL	MD BL	MD BL	MD BL	MD LL/BL

Average	8.5 7.0	6.5 6.0	7.5 8.0	7.0 9.0	7.0 9.0	10.0 11.0	9.0 11.0	55.5 61.0

Patient	7.5 6.1	4.7 5.6	6.9 7.4	6.4 7.8	6.5 7.7	9.5 10.4	8.7 10.9	50.2 55.9

Mandible	MD LL	MD LL	MD LL	MD BL	MD BL	MD BL	MD BL	MD LL/BL

Average	5.0 6.0	5.5 6.5	7.0 7.5	7.0 7.5	7.0 8.0	11.0 10.5	10.5 10.0	53.0 56.0

Patient	4.4 5.6	5.2 5.8	6.6 7.1	6.7 7.3	6.8 7.7	10.7 10.3	9.9 9.4	50.3 53.2

Measurements in millimeters were taken at widest portion of clinical crown on diagnostic casts. *Anatomic average taken from Wheeler, R. C.: Textbook of Dental Anatomy and Physiology, ed. 7, Philadelphia, 1993, W. B. Saunders Company, pp. 25.

**Table 2:** Comparison of buccolingual/labiolingual and mesiodistal crown dimensions with an anatomic average* of the left side maxillary and mandibular teeth

Left side	Central incisor	Lateral incisor	Canine	First premolar	Second premolar	First molar	Second molar	Total
Maxillary	MD LL	MD LL	MD LL	MD BL	MD BL	MD BL	MD BL	MD LL/BL

Average	8.5 7.0	6.5 6.0	7.5 8.0	7.0 9.0	7.0 9.0	10.0 11.0	9.0 11.0	55.5 61.0

Patient	7.2 5.8	4.5 5.4	7.1 7.5	6.2 7.6	6.3 7.7	9.3 10.2	8.7 10.9	49.3 52.1

Mandible	MD LL	MD LL	MD LL	MD BL	MD BL	MD BL	MD BL	MD LL/BL

Average	5.0 6.0	5.5 6.5	7.0 7.5	7.0 7.5	7.0 8.0	11.0 10.5	10.5 10.0	53.0 56.0

Patient	4.2 5.7	5.3 6.1	6.7 7.3	6.7 7.2	6.8 7.7	10.7 10.2	9.6 8.9	50.0 53.1

Measurements in millimeters were taken at widest portion of clinical crown on diagnostic casts. *Anatomic average taken from Wheeler, R. C.: Textbook of Dental Anatomy and Physiology, ed. 7, Philadelphia, 1993, W. B. Saunders Company, pp. 25.

The simultaneous presence of microdontia and supernumery teeth is been reported in the Cleidocranial dysplasia, Craniometadiaphyseal dysplasia, Dermoodontodysplasia, Hypodontia and nail dysgenesis, Orofaciodigital syndrome type 3 and Tricho-rhino-phalangeal syndrome type 1. However in this case, except for the dental abnormality in the form of generalized microdontia and the presence of fully erupted mandibular mesiodens between the central incisors were found and no other clinical features observed, therefore all the syndrome associated with the simultaneous presence of microdontia and supernumery teeth were ruled out along with Taurodontism, microdontia, and dens invaginatus as well as Distal symphalangism, hypoplastic carpal bones, microdontia, dental pulp stones, narrowed zygomatic arch (Table 3).

**Table 3:** Comparison of conditions associated with the simultaneous presence of microdontia and supernumery teeth along with taurodontism, microdontia, and dens invaginatus as well as distal symphalangism, hypoplastic carpal bones, microdontia, dental pulp stones, narrowed zygomatic arch.

Taurodontism, microdontia, and dens invaginatus	Cleidocranial dysplasia	Craniometadiaphyseal dysplasia	Dermoodontodysplasia	Hypodontia and nail dysgenesis	Orofaciodigital syndrome type 3	Tricho-rhino-phalangeal syndrome type 1	Distal symphalangism, hypoplastic carpal bones, microdontia, dental pulp stones, narrowed zygomatic arch
Generalized microdontia	Autosomal dominant	Autosomal recessive	Autosomal dominant	Autosomal dominant	Autosomal recessive	Autosomal dominant	Autosomal dominant
Taurodontism of first permanent molars	Chromosome 6	Macrocephaly	Dry skin	Chromosome 4	Thin/hyperconvex/hypoplastic nails	Autosomal recessive	Absent/small nails
Multiple teeth with one or more dens invaginatus	Arm p	Frontal bossing	Ichthyosis	Arm p	Prominent occiput	Chromosome 8	Microdontia
X-linked recessive inheritance	Normal height (with skeletal dysplasia)	Large fontanelle	Thin skin	Dry skin	Frontal bossing	Arm q	Other dental abnormality
	Short stature - postnatal	Prominent eyes	Pigmented naevi	Fine hair	Round face	Normal height (with skeletal dysplasia)	Abnormal clinical features of the limbs
	Absent/small nails	Mandibular hyperostosis/sclerosis	Abnormal hair texture	Brittle hair/trichorrhexis nodosa/pili torti	Hypertelorism	Short stature - postnatal	Brachydactyly
	Macrocephaly	Optic nerve abnormality/atrophy	Sparse/absent scalp hair - localised	Sparse/absent scalp hair - generalised	Down-slanting palpebral fissures	Decreased body hair/hypotrichosis	Irregularities of length/shape of fingers
	Flat occiput (brachycephaly)	Microdontia	Abnormal nails	Absent/small nails	Other orbital abnormality	Decreased hair pigmentation - general	Syndactyly of fingers
	Frontal bossing	Abnormal tooth position/malocclusion/open bite	Midface hypoplasia/flat/short midface	Thin/hyperconvex/hypoplastic nails	Paresis of ocular muscles/squint	Decreased hair pigmentation - patchy	Short foot (including brachydactyly)
	Wide sutures/delayed fusion of sutures	Missing permanent teeth/retained deciduous teeth	Micrognathia/agnathia/retrognathia	Dysplastic/grooved/thick/discoloured nails	Other eye movement disorder	Fine hair	Syndactyly (other than minimal 2nd and 3rd toes)
	Large fontanelle	Anodontia/oligodontia	Microdontia	Depressed premaxillary region	Broad/bulbous nasal tip	Brittle hair/trichorrhexis nodosa/pili torti	Irregular length or shape of toes
	Facies significantly abnormal	Natal/neonatal teeth	Anodontia/oligodontia	Midface hypoplasia/flat/short midface	Cleft soft palate/bifid uvula/submucous cleft	Sparse/absent scalp hair - generalised	Other skull abnormality
	Small face	Supernumerary teeth	Supernumerary teeth	Micrognathia/agnathia/retrognathia	Microdontia	High hairline - front	Absent/small/hypoplastic carpals
	Hypertelorism	Dental caries	Other dental abnormality	Absent/decreased eyebrows/lateral thinning	Abnormal tooth position/malocclusion/open bite	Thin/hyperconvex/hypoplastic nails	Symphalangism
	Prominent supraorbital ridges	Low set ears	Simian creases	Absent/decreased lashes	Supernumerary teeth	Dysplastic/grooved/thick/discoloured nails	Cone shaped epiphyses
	Depressed premaxillary region	Scoliosis	Dislocated hip	Everted/protruding lips	Cleft/notched tongue	Broad/bifid nails	Symphalangism
	Midface hypoplasia/flat/short midface	Bowed limbs		Tooth crown shape abnormality	Hamartoma/other tumours of the mouth	Frontal bossing	Cone-shaped epiphyses of middle phalanges
	Prognathism	Mental retardation of any degree		Microdontia	Other abnormality of tongue/gingivae/mucosa	High forehead	
	Depressed nasal bridge	Boney sclerosis of any type		Abnormal tooth position/malocclusion/open bite	Low set ears	Facies significantly abnormal	
	Paramedian/lateral cleft lip (uni/bilateral)	Multiple fractures/increased boney fragility		Delayed eruption of teeth	Tragus abnormal	Long face	
	Cleft soft palate/bifid uvula/submucous cleft	Enchondroma/radiolucencies - localized		Anodontia/oligodontia	Pectus excavatum (funnel chest)	Grooved/dimpled chin	
	High vaulted and narrow palate	Lytic/lucent lesions of bone		Supernumerary teeth	Abnormally placed nipples	Micrognathia/agnathia/retrognathia	
	Microdontia	Fibrous dysplasia of bone			Thoracolumbar general kyphosis	Medial flare of eyebrows	
	Developmental defect of enamel	Wide diaphyses (undertubulation)			Irregularities of length/shape of fingers	Absent/decreased eyebrows/lateral thinning	
	Tooth discolouration	Submetaphyseal undermodelling/expansion			Syndactyly of fingers	Absent/decreased lashes	
	Delayed eruption of teeth	Thin cortex of diaphyses			Polydactyly - postaxial (ulnar)/type unspecified	Long/large nose	
	Missing permanent teeth/retained deciduous teeth	Bowing of long bones			Abnormal palmar dermatoglyphics/skin creases	Broad nasal bridge (see telecanthus)	
	Supernumerary teeth	Cartilage tongues of metaphyses - localized			Polydactyly of feet - postaxial/type unspecified	High nasal bridge	
	Dental cysts/tumours	Hyperostosis/thickened/sclerotic calvarium			Syndactyly (other than minimal 2nd and 3rd toes)	Broad/bulbous nasal tip	
	Deafness - conductive	Absent/abnormal sinuses			Cranial nerve/nuclei	Hypoplastic/small nostrils	
	Other hearing abnormality	Wormian bones			Mental retardation - moderate/severe	Abnormal columella	
	Narrow/sloping shoulder/hypermobile shoulders	Sclerotic/hyperostotic facial bones			Hypotonia	Thin lips	
	Pectus excavatum (funnel chest)	Other skull abnormality			Movement disorder - dystonia/chorea/tremor/spasm	Long philtrum	
	Bell-shaped chest	Hyperostotic/wide clavicle			EEG abnormality	Deeply grooved philtrum	
	Thoracolumbar general kyphosis	Abnormal rib structure including fusion			Short sternum	Microdontia	
	Gibbus/localised kyphosis	Widened ribs				Abnormal tooth position/malocclusion/open bite	
	Scoliosis	Irregular shape of pubic and ischial bones				Supernumerary teeth	
	Hyperextensible/hypermobile joints	Absent/hypoplastic/short femur				Anteverted/prominent/bat ears	
	Small hand	Femora short/deformed/bowed				Long/large ear	
	Brachydactyly	Other abnormal femur				Pectus carinatum (pigeon chest)	
	Seizures of any type	Bow legs - genu varum				Thoracolumbar general kyphosis	
	Hypotonia					Scoliosis	
	Imperforate anus/anal stenosis					Hyperextensible/hypermobile joints	
	Horseshoe/fused/ectopic kidneys					Small hand	
	Hypospadias/epispadias					Brachydactyly	
	Undescended/ectopic testes					Clinodactyly of 5th finger	
	Wilms tumour					Terminal hypoplasia fingers	
	Delayed skeletal maturation					Spindle shaped/tapered fingers	
	Poorly ossified calvarium/Soft skull					Ulnar deviation of fingers	
	Absent/abnormal sinuses					Other hand abnormality	
	Wormian bones					Mental retardation of any degree	
	Platybasia/basilar impression					Abnormal cardiovascular structure/function	
	Enlarged foramen magnum					Winged/other abnormal scapula (See Shoulder)	
	Small/absent scapula					Coxa vara	
	Winged/other abnormal scapula (See Shoulder)					Cone shaped epiphyses	
	Absent/hypoplastic clavicles					Small femoral head epiphyses	
	Pseudarthrosis of clavicle					Flat femoral head epiphyses	
	Short ribs (circumferential)					Deformed/irregular femoral head epiphyses	
	Under-/unossified sternum					Broad femoral neck	
	Hypoplastic/absent ribs					Cone-shaped epiphyses of proximal phalanges	
	Dorsal wedging of vertebral bodies					Some phalanges short and deformed	
	Narrow/trapezoid iliac wings (lack of flare)					Cone-shaped epiphyses of middle phalanges	
	Horizontal/flat acetabular roof					Cone-shaped epiphyses of distal phalanges	
	Delayed ossification of pubic and ischial bones						
	Open pubic symphysis in adults						
	Coxa valga						
	Coxa vara						
	Dislocated hip						
	Cone shaped epiphyses						
	Fibulae a-/hypoplastic/under-/unossified						
	Cone-shaped epiphyses of proximal phalanges						
	Cone-shaped epiphyses of middle phalanges						
	All middle phalanges short/deformed						
	Cone-shaped epiphyses of distal phalanges						
	All distal phalanges short/deformed						

A diagnosis of non-syndromic occurance of true generalized microdontia with mandibular mesiodens was made as no systemic condition was observed. The fully erupted mandibular mesiodens was extracted under local anesthesia in order to correct midline and to facilitate the orthodontic treatment.

## Discussion

The initiating factor or factors responsible for microdontia remain obscure. Mutation in developmental regularity genes are known to cause variety of dental defects [16]. Both genetic and environmental factors are involved in the complex etiology of microdontia. Genetic factors probably play a role in the formation of microdontia. Although the proband was the only child, the presence of consanguinity in the form of both parents being maternal first cousins could suggest recessive or polygenic inheritance.

The development of a tooth has been shown to have ectodermal, mesodermal, and neural crest contributions. The variation in size of a particular tooth arises during the period when the form of the tooth is being determined by the enamel organ and the sheath of hertwig at the bell stage of enamel organ. The determination of the form of the crown is thought to be related to different regions of the oral epithelium or to the ectomesenchyme. Studies have shown that different regions of the oral epithelium rather than the underlying ectomesenchyme are initially responsible for the shape of the crown [17]. Bones dating from the Middle Ages which were excavated at Alborg, Denmark proved evidence for generalized microdontia resulting from intrauterine growth retardation [18].

On the basis of visual documentation, the patient in the current case seems to have been more severely affected in all his teeth which exhibited aberrant morphology and all were smaller than normal. MEDLINE search in the English dental literature for true generalized microdontia revealed zero search results. Although child's mother had difficult delivery, it was insignificant and neither microdontia nor mesiodens has been reported in the literature.

The prevalence of mesiodens varies between 0.09 and 2.05% in different studies. In permanent dentition, a 0.15 to 3.8% incidence of mesiodens has been reported [19]. Erupted supernumerary teeth in the mandible are rare, is about 0.01% which indicated marked low value [20]. Supernumerary teeth in the mandible anterior region in this case is fully erupted which is unusual.

Sexual dimorphism is reported by most authors with males being more commonly affected. Hogstrum and Andersson [21] reported a 2:1 ratio of sex distribution. A study of supernumerary teeth in Asian school children found a greater male to female distribution of 6.5:1 for Hong Kong children [22] which indicates that supernumery teeth is more common in males than females which is consistent in our case.

Non-syndromic multiple supernumerary teeth occur most frequently in the mandible region especially premolar region followed by molar and anterior region [9]. Few cases of non-syndrome multiple supernumery teeth have been reported [23,24] however in the present case non-syndromic single supernumerary tooth was observed in the mandibular anterior region.

Evidence regarding etiology of mesiodens indicates that genetic susceptibility together with environmental factors might increase the activity of dental lamina leading to formation of the extra tooth/teeth [19]. A number of theories have been proposed as regards the causes of the occurrence of supernumerary teeth: 1] Atavism theory [8,24,25] 2] Independent hyperactivity of the dental lamina [24,25] and 3] Dichotomy of the tooth bud are also suggested as a possible etiological factors [8,25]. However, none of these theories alone offers a sufficient explanation for this phenomenon.

Since mesiodens may interfere with normal occlusal development, in the present case an early diagnosis could have prevented the lower diastema formation. Early diagnosis and treatment of patients with supernumerary teeth are important to prevent or minimize complications.

As the patient did not show any abnormal systemic manifestations, all the syndrome associated with the dental anomalies were ruled out. The simultaneous presence of supernumerary teeth and the generalized microdontia is very rare. To our knowledge, this is the first such case of non-syndromic occurance of true generalized microdontia in association with mandibular mesiodens. Such unusual nature of dental anomaly has not been reported so far in the literature.

## Conclusion

The dental finding seen in this case is certainly rare. The case is also sporadic, with no positive family history. The wide variation in clinical manifestations in cases of non-syndromic occurrence of dental anomalies is challenging and is an area for further research. Mesiodens are familiar to pediatric dentists and orthodontists as one of the more common anomalies to affect the developing dentition and it demands a multidisciplinary assessment.

## Consent

Written informed consent was obtained from the patient for publication of this Case report and any accompanying images. A copy of the written consent is available for review by the Editor-in-Chief of this journal.

## Competing interests

The authors declare that they have no competing interests.

## Authors' contributions

SB and SK drafted the manuscript paper, analysed the patient's history and contributed to the writing of the final version as well as extracted the mesiodens. Each author reviewed the paper for content and contributed to the writing of the manuscript. All authors approved the final report.

## References

[B1] Laundau S International dictionary of medicine and biology 1986 1 New York: John Wiley & Sons 1717

[B2] Boyle PE Kronfeld's Histopathology of the Teeth and their Surrounding Structures 1955 3 Philadelphia: Lea& Febiger 14

[B3] Shafer WG Hine MK Levy BM A Textbook of Oral Pathology 1958 1 Philadelphia: W. B. Saunders Co 26

[B4] Ufomata D Microdontia of a mandibular second premolar Oral Surg Oral Med Oral Pathol 1988 65 637 8 10.1016/0030-4220(88)90150-8 3163793

[B5] Van der waal I Van der kwast WAM Developmental anomalies and eruption disturbances and some acquired disorders of the teeth Oral pathology 1988 Chicago: Quintessence Publishing Co. Inc 114

[B6] Opinya GN Kaimenyi JT Meme JS Oral findings in Fanconi's anemia: a case report J Periodontol 1988 59 461 463 3166059 10.1902/jop.1988.59.7.461

[B7] Schulze C Gorlin RJ, Goldman HM Developmental abnormalities of the teeth and jaws Thoma's Oral Pathology 1970 St Louis: Mosby 112 2222007233

[B8] Rajab LD Hamdan MA Supernumerary teeth: review of the literature and a survey of 152 cases Int J Paediatr Dent 2002 12 244 54 10.1046/j.1365-263X.2002.00366.x 12121534

[B9] Bhat M Supplemental mandibular central incisor J Indian Soc Pedod Prev Dent 2006 24 Suppl 2 s20 s23 16891745

[B10] Tanaka S Murakami Y Fukami M Nakano K Fujisawa S Miyoshi S A rare case of bilateral supernumerary teeth in the mandibular incisors Br Dent J 1998 185 386 8 10.1038/sj.bdj.4809822 9828497

[B11] Fukuta Y Totsuka M Takeda Y Yamamoto H Supernumerary teeth with eumorphism in the lower incisor region:a report of five cases and review of the literature J Oral Sci 1999 41 199 202 10693298 10.2334/josnusd.41.199

[B12] Alencar M Duarte D Cury P Bonecker M Lower mesiodens: report of an unusual case J Clin Pediatr Dent 2005 29 353 356 16161403 10.17796/jcpd.29.4.u1j5249mv7p288l6

[B13] Yokose T Sakamoto T Sueishi K Yatabe K Tsujino K Kubo S Yakushiji M Yamaguchi H Two cases with supernumerary teeth in lower incisor region Bull Tokyo Dent Coll 2006 47 19 23 10.2209/tdcpublication.47.19 16924155

[B14] Grga D Dzeletovic B Supernumerary Tooth in Lower Incisor Region: A Case Report Serbian Dental Journal 2010 57 220 222

[B15] Zhu JF Marcushamer M King DL Henry RJ Supernumerary and Congenitally absent teeth: a literature review J Clin Pediat Dentist 1996 20 87 95 8619981

[B16] Thesleff I Genetic basis of development of dental defects Acta Odontol Scand 2000 58 191 4 10.1080/000163500750051728 11144868

[B17] Osborn JW Ten Cate AR Advanced dental histology: dental practitioner. Handbook no, 6 1976 3 Bristol: John Wright & Sons 24 51

[B18] Alexandersen V Nielsen OV Generalized microdontia probably associated with intrauterine growth retardation in a medieval skeleton American Journal of Physical Anthropology 1970 33 389 401 10.1002/ajpa.1330330313 4321404

[B19] Meighani G Pakdaman A Diagnosis and Management of Supernumerary (Mesiodens): A Review of the Literature Journal of Dentistry 2010 7 41 49 21998774 PMC3184724

[B20] Stafne EC Supernumerary teeth Dental Cosmos 1932 74 653 659

[B21] Hogstrom A Andersson L Complications related to surgical removal of anterior supernumerary teeth in children ASDC J Dent Child 1987 54 341 3 3478360

[B22] Davis PJ Hypodontia and hyperodontia of permanent teeth in Hong Kong school children Community Dentistry and Oral Epidemiology 1987 15 218 20 10.1111/j.1600-0528.1987.tb00524.x 3476247

[B23] Srivatsan P Aravindha Babu N Mesiodens with an unusual morphology and multiple impacted supernumerary teeth in a non-syndromic patient Indian J Dent Res 2007 18 138 40 10.4103/0970-9290.33792 17687179

[B24] Sivapathasundharam B Einstein A Non-syndromic multiple supernumerary teeth: Report of a case with 14 supplemental teeth Indian J Dent Res 2007 18 144 10.4103/0970-9290.33794 17687181

[B25] Stellzig A Basdra EK Komposch G Mesiodentes: incidence, morphology, etiology J Orofac Orthopaedic 1997 58 144 153 10.1007/BF02676545 9200890

